# Supplementary material for: Biomarker Analysis from a Phase I/Ib Study of Regorafenib and Nivolumab in Mismatch Repair-Proficient Advanced Refractory Colorectal Cancer
Source: Cancers (Basel). 2024 Jan 28;16(3):556. doi: 10.3390/cancers16030556 (PMC10854756; doi:10.3390/cancers16030556)
Supplement: Supplementary file 1 [file cancers-16-00556-s001.zip › cancers-2825415-supplementary.pdf]

Supplement Figure S1

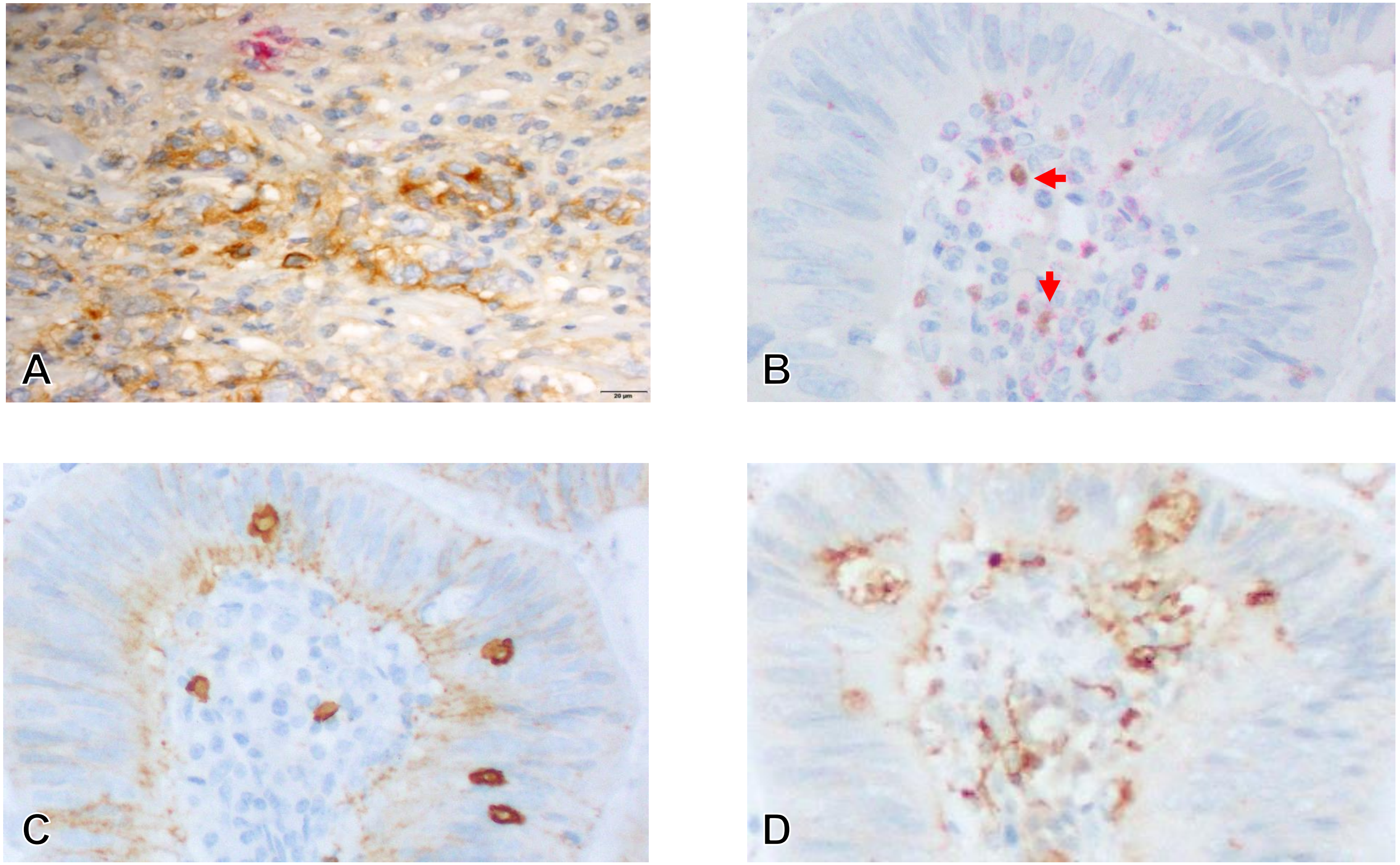

Representative immunohistochemial expression of PD-1, PD-L1, CD4, FOXP3, CD8 and CD68. A: PD-1 (red chromogen) and PD-L1 (brown chromogen) B: CD4 (red chromogen), FOXP3 (brown chromogen), dual expression of CD4 and FOXP3 (red arrows), C: CD8 (brown chromogen), D: CD68 (brown chromogen).

Supplement Figure S2

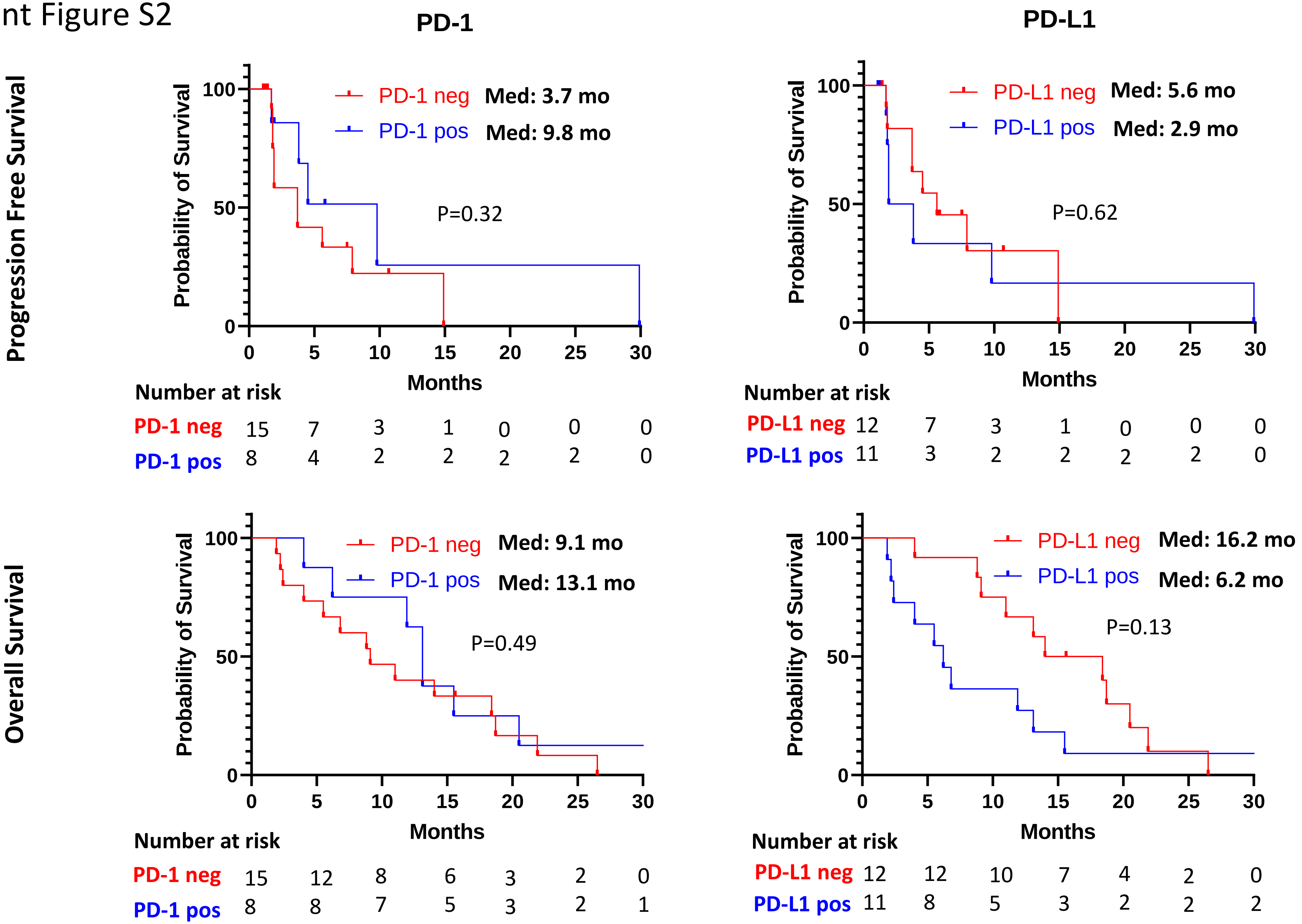

Kaplan-Meier estimates of progression free survival and overall survival by PD-1 and PD-L1 expression in tumor microenvironment.

Supplement Figure S3

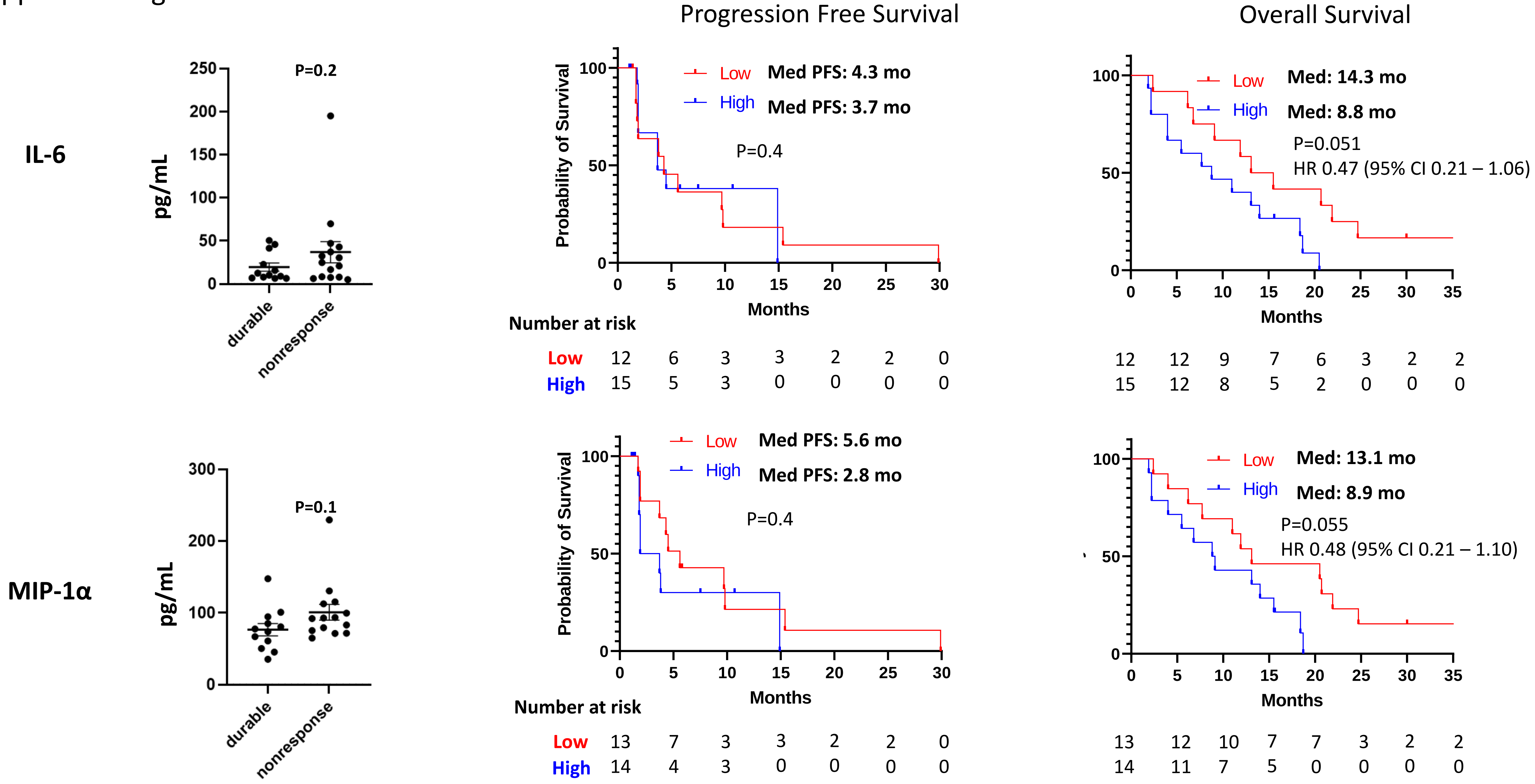

Baseline IL-6 and MIP-1α level between durable responders and non-responders. Kaplan-Meier estimates of progression free survival and overall survival by IL-6 and MIP-1α.

Supplement Figure S4

Heat Map Analysis

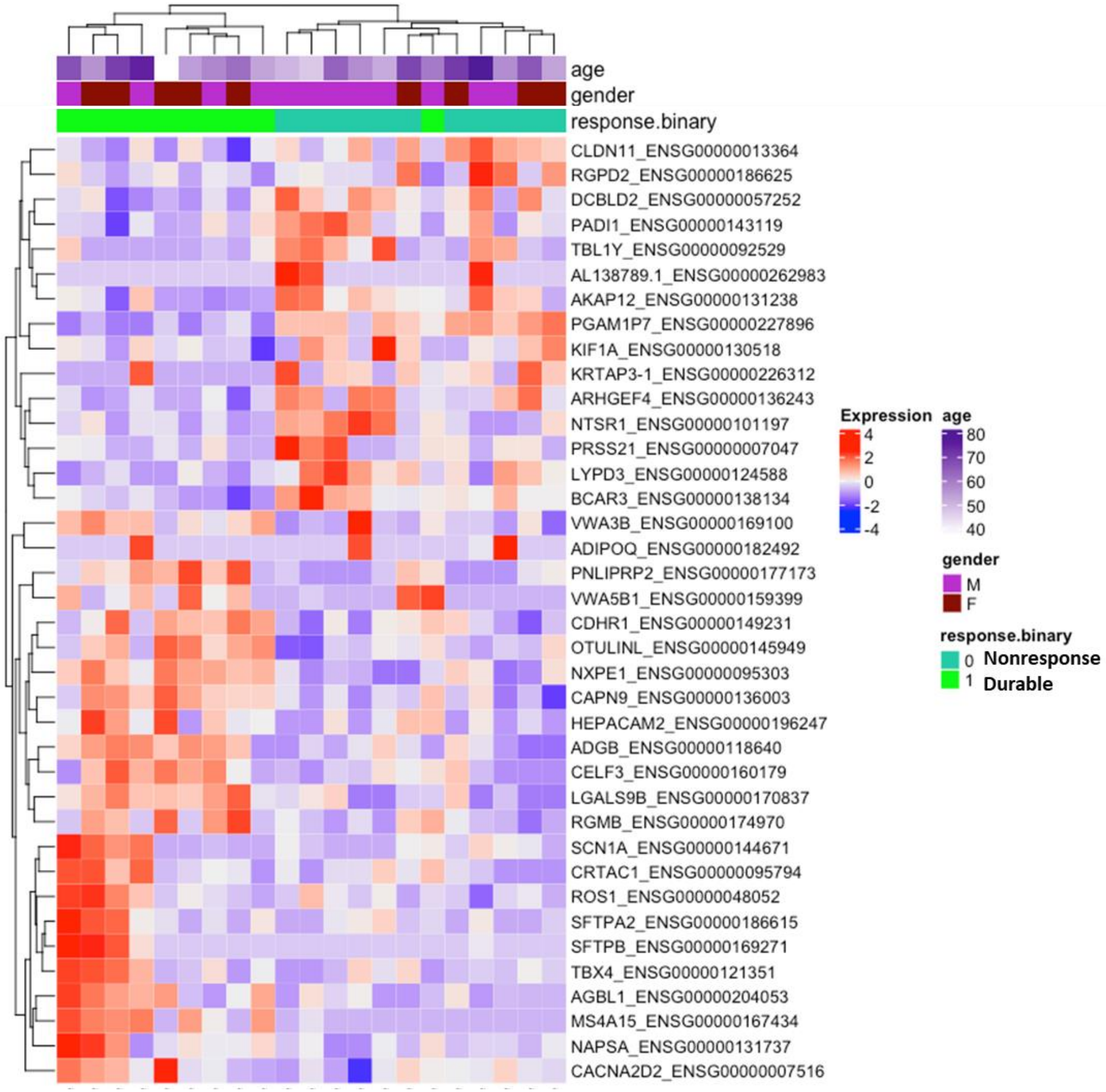

Heat map analysis of gene expression profile. Rows represent genes and columns represent patients. The first row indicates age, then gender and response.

Supplement Figure S5

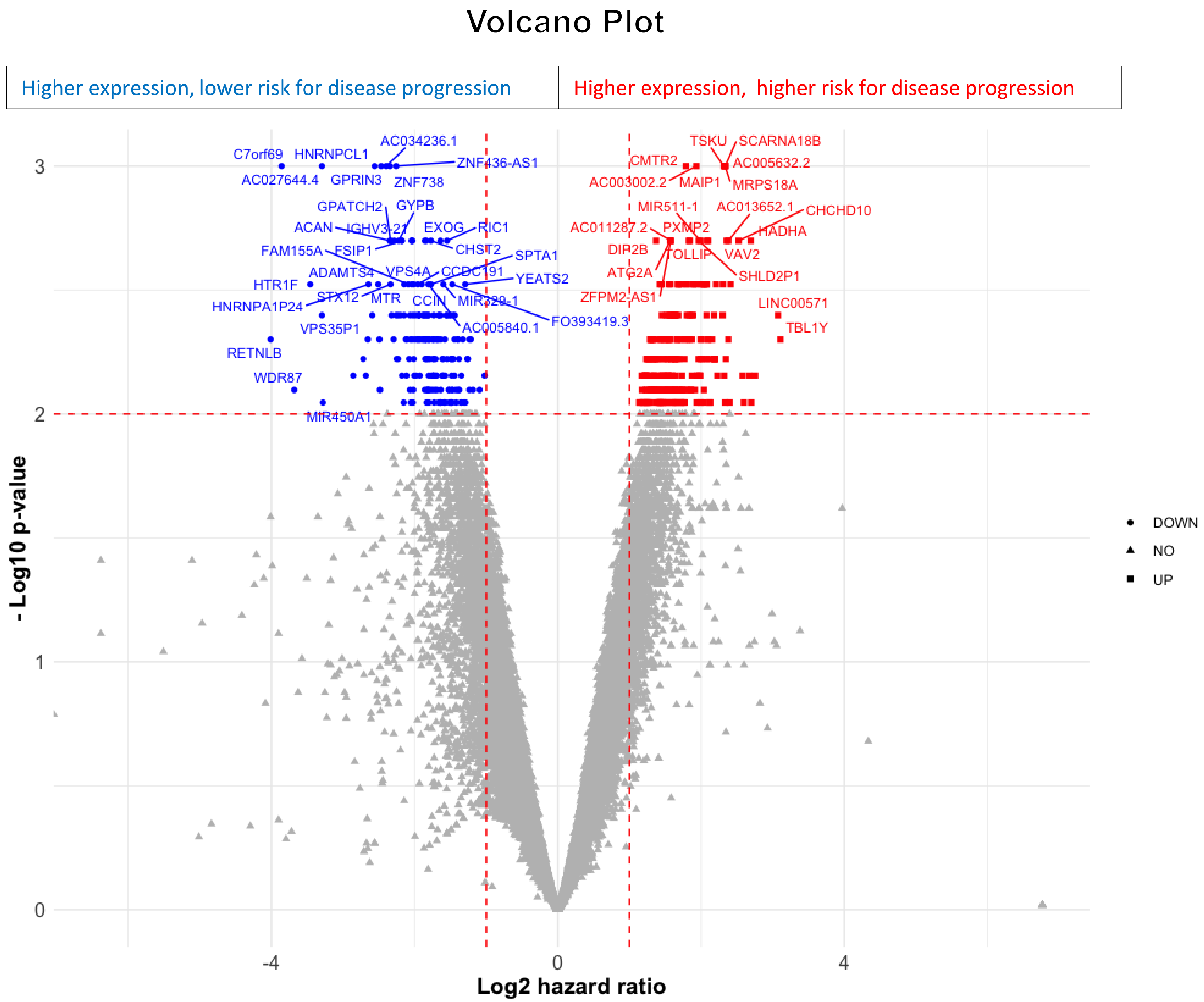

Volcano plot of differential expressed genes expression profile associated with disease progression.

Supplement Table S1

	Non-liver (N=14)	Liver (N=37)	P value
**Albumin**			
Low	1 (7.1%)	13 (35.1%)	P= 0.077
High	13 (92.9%)	24 (64.9%)	

The correlation between baseline levels of albumin and the presence or absence of metastatic liver disease

Supplement Table S2

	Non-liver (N=6)	Liver (N=17)	P value
**CD8**			
Low	2 (33.3%)	9 (5.29%)	P= 0.4
High	4 (66.7%)	8 (47.1%)	
**Treg**			
Low	5 (83.3%)	9 (52.9%)	P= 0.2
High	1 (16.7%)	8 (47.1%)	
**CD68**			
Low	3 (50%)	7 (41.2%)	P= 0.7
High	3 (50%)	10 (58.8%)	
**CD163**			
Low	5 (83.3%)	8 (47.1%)	P=0.1
High	1 (16.7%)	9 (52.9%)	

Frequency of CD8, Treg (regulatory T cells), CD68 and CD163 in the metastatic sites (non-liver and liver metastatic site)

Supplement Table S3

Marker	Unit	Median (range)
IL-13	pg/mL	7.4 (5.3 - 10.9)
IL-15	pg/mL	15.8 (11.8 - 24.9)
IL-17A	pg/mL	6.3 (3.1 - 29.5)
IL-18	pg/mL	3051.6 (1522.8 - 6102.9)
IL-2	pg/mL	1.9 (1.6 - 3.5)
IL-4	pg/mL	0.2 (0.2 - 0.8)
IL-5	pg/mL	3.3 (1.0 - 10.0)
IL-6	pg/mL	20.9 (5.2 - 195.1)
IL-7	pg/mL	56.6 (26.0 - 105.3)
IL-8	pg/mL	153.3 (33.6 - 3135.9)
IP-10	pg/mL	1712.8 (512.5 - 4054.3)
MCP-1	pg/mL	1427.1 (803.8 - 2143.8)
MIP-1α	pg/mL	92.1 (35.4 - 1148.7)
MIP-1β	pg/mL	313.6 (137.4 - 722.7)
TNF-α	pg/mL	2.8 (0.9 - 6.2)
VEGF-A	pg/mL	138.1 (40.1 - 994.7)

Baseline levels of 16 cytokines and chemokines with the median level (range)
